# The Role of Paxillin Aberrant Expression in Cancer and Its Potential as a Target for Cancer Therapy

**DOI:** 10.3390/ijms24098245

**Published:** 2023-05-04

**Authors:** Weixian Liu, Xinxian Huang, Weizhao Luo, Xinguang Liu, Weichun Chen

**Affiliations:** 1 Guangdong Provincial Key Laboratory of Medical Molecular Diagnostics, Institute of Aging Research, Guangdong Medical University, Dongguan 523808, China; 2 Institute of Biochemistry and Molecular Biology, Guangdong Medical University, Zhanjiang 524023, China

**Keywords:** paxillin, aberrant expression, metastasis, mechanism, treatment target

## Abstract

Paxillin is a multi-domain adaptor protein. As an important member of focal adhesion (FA) and a participant in regulating cell movement, paxillin plays an important role in physiological processes such as nervous system development, embryonic development, and vascular development. However, increasing evidence suggests that paxillin is aberrantly expressed in many cancers. Many scholars have also recognized that the abnormal expression of paxillin is related to the prognosis, metastases, invasion, survival, angiogenesis, and other aspects of malignant tumors, suggesting that paxillin may be a potential cancer therapeutic target. Therefore, the study of how aberrant paxillin expression affects the process of tumorigenesis and metastasis will help to develop more efficacious antitumor drugs. Herein, we review the structure of paxillin and its function and expression in tumors, paying special attention to the multifaceted effects of paxillin on tumors, the mechanism of tumorigenesis and progression, and its potential role in tumor therapy. We also hope to provide a reference for the clinical prognosis and development of new tumor therapeutic targets.

## 1. Introduction

Paxillin is a 68 kDa multi-domain adaptor protein that is a major constituent of focal adhesions (FAs) and is expressed in virtually all tissues [1]. As a signaling scaffold, paxillin can recruit a variety of intracellular signaling molecules to bind to various proteins. However, interestingly, paxillin itself has no enzymatic activity, and it is post-translationally modified to be biased towards a specific set of conformations. This allows it to interact with different proteins to transduce extracellular signals triggered by the binding of integrins to the extracellular matrix (ECM) into cells to cause various signaling cascades, which play different roles in tissue development stages and specific physiological processes. Importantly, paxillin is also involved in pathological processes such as oxidative stress, inflammation, and endothelial cell barrier dysfunction [2,3]. Although paxillin is primarily localized in FA, it is also present in the nucleus, where it is thought to act as a transcription factor affecting the transcription of certain genes [4].

However, the special structure and function of paxillin make it play an indispensable role in the development of cancer. Growing evidence has shown that paxillin is aberrantly expressed in a variety of human malignancies, such as melanoma [5], breast cancers [6], gastric cancers [7], and colorectal cancers [8]. Additionally, the expression of paxillin is associated with the poor prognosis, occurrence, and metastasis of tumors. Therefore, paxillin may be a potential target for the development of drugs to treat tumor growth and metastasis. In this review, we will briefly introduce the expression of paxillin in human cancer, its prognostic value, and how it affects malignant tumor progression and molecular mechanism, as well as the evidence contributing towards its potential to be used as a therapeutic target.

## 2. Structure and Expression of Paxillin in Tumors

### 2.1. Structure of Paxillin

**Figure 1:**
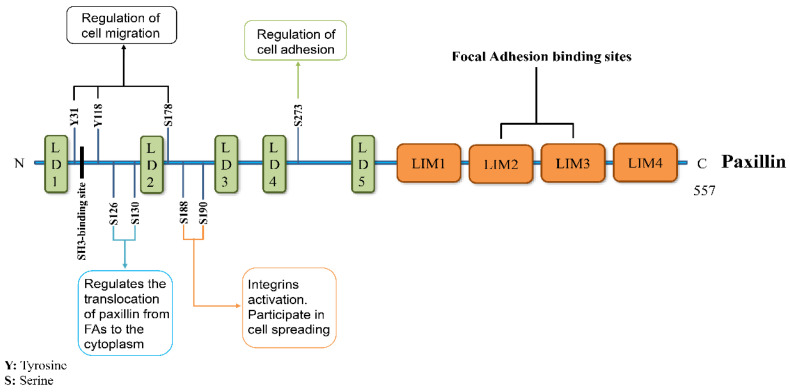
Paxillin structure and function. Paxillin consists of 557 amino acids, of which the N-terminus contains five LD domains for recruiting signaling molecules and a proline-rich region responsible for binding to SH3-containing proteins. The C-terminus contains four cysteine–histidine-rich LIM domains, of which LIM2 and LIM3 are responsible for targeting paxillin to focal adhesions. Some tyrosine and serine phosphorylation sites on the structure of paxillin can bind to many signaling molecules so that paxillin participates in cell migration and movement and regulates paxillin targeting FA.

Paxillin mainly consists of five leucine–aspartic acid (LD) domains at the N-terminal, four cysteine–histidine-rich Lin11, Isl-1, and Mec-3 (LIM) domains at the C-terminal, and a proline-rich sequence and some serine and tyrosine residues, in which the N-terminal LD domain controls most of its signaling activities (Figure 1). LD motifs form amphipathic α-helices, while negatively charged aspartate and glutamate residues flank leucine residues on the helical face to form hydrophobic patches [9]. Meanwhile, the N-terminus of paxillin also contains a proline-rich region, which exists between LD1 and LD2, which was initially thought to bind to the SH3 domain of the non-receptor tyrosine kinase Src and was later proven to bind the vinculin-binding protein poison which interacts to form a polyproline-II helix [10]. The LD domain contains many phosphorylation sites that play a role in protein activation and signal transduction so that it can bind to a variety of FA-related proteins, such as the Src family of tyrosine kinases, focal adhesion kinases (FAKs), integrin-linked kinase, p21-activated kinase, vinculin, and talin. [3].

Studies have shown that p21-activated kinase can phosphorylate paxillin at tyrosine residue 273, which increases the cell migration ability and neurite activity and makes paxillin a regulator of cell adhesion [11]. The phosphorylation of paxillin at serine residue 178 by c-jun N-terminal kinase (JNK) is also required for cell migration [12]. Activated integrins can also induce the phosphorylation of paxillin at tyrosine 31 and tyrosine 118 to promote the migration of certain cells [13,14,15]. At the same time, integrins can also bind to the extracellular matrix (ECM) to activate the phosphorylation of paxillin at serine residues 188 and serine residues 190 to induce cell proliferation [16]. In addition, glycogen synthase kinase 3 and the activated MAPK/ERK pathway can, respectively, induce paxillin phosphorylation at serine residues 130 and serine residues 126, which directs paxillin to participate in cell spreading and translocation from FA to the cytoplasm [17,18].

Despite the fact that the LD domain can bind to a variety of FA-related proteins, the real targeting of paxillin to FA is the four cysteine–histidine-rich LIM domains at the C-terminal of paxillin. All four LIM domains are double zinc finger motifs that anchor paxillin to the plasma membrane and can mediate protein–protein interactions [19,20]. Among the LIM domains, the LIM2 and 3 domains are responsible for targeting paxillin to FA, and the phosphorylation of these domains regulates the relocalization of paxillin in FA, but in fact, the primary mechanism for targeting paxillin to FA is utilized through LIM3 [21]. Meanwhile, the specific binding of individual paxillin LIM2 and 3 domains to tubulin may play a role in regulating the subcellular compartmentalization of paxillin [22].

### 2.2. Expression of Paxillin in Tumors

The expression of paxillin plays an important role in the human body both in the process of physiological development and in the process of disease development. Although paxillin mutations in somatic cells are rare, studies have also shown that paxillin mutations are associated with tumor progression. In a study of lung cancer, the expression of paxillin in cancer tissue was higher than in normal lung tissue, and the somatic mutation rate was 9.4%, of which the most common point mutation (A127T) enhanced the growth and invasion ability of lung cancer cells [23]. Later, as more and more studies showed that the abnormal expression of paxillin was associated with the malignant progression of tumors (Table 1), Chen et al. evaluated the high expression of the paxillin gene and protein in glioma tissue using the immunohistochemical method to contribute to the malignant progression of tumor and proved that the high expression of paxillin is significantly correlated with the malignant degree of glioma [24]. Paxillin-mediated ERK activation using the phosphorylating Bcl-2 protein to increase its stability was shown to be responsible for paxillin-mediated cancer cell invasiveness [25]. Similarly, paxillin has been shown to be highly expressed in gastric cancer tissues and cell lines; patients with a high paxillin expression not only have a poor prognosis, but this high expression also promotes proliferation and migration in vitro [7].

Paxillin is phosphorylated at the tyrosine-88 residue (Y88-paxillin) by the oncogenic kinase Src to form pY88-paxillin, which is highly expressed in most human colon cancer tissues, and pY88-paxillin plays an oncogenic role in colorectal tumorigenesis [26]. Additionally, the mRNA and protein levels of paxillin were also higher in high metastatic potential human breast cancer cells than in low metastatic potential cells [6]. In a recent study, pan-cancer analysis revealed that paxillin expression is not necessarily the same in different tumor types. The expression of paxillin in human cholangiocarcinoma, esophageal cancer, hepatocellular carcinoma, and other tumors is significantly higher than in paired normal tissues, but the expression in several tumors, such as renal papillary cell carcinoma and endometrial carcinoma, is opposite. Additionally, a higher paxillin expression is generally associated with poorer overall survival and disease-free survival, suggesting that paxillin has multiple regulatory mechanisms in different tumor types [27]. Overall, there is a consistent conclusion that can be made: aberrant paxillin expression is associated with clinical tumor prognosis, proliferation, invasion, and metastasis.

**Table 1:** Expression patterns of Paxillin in different cancers.

System	Tumor Type	Expression	Level	Reference
Nervous system	Glioma	Upregulated	Tissue	[24]
Digestive system	GC	Upregulated	Tissue and cell	[7]
	CRC	Upregulated	Tissue	[8]
	Esophageal cancer	Upregulated	Tissue	[28]
	LSCC	Upregulated	Tissue	[29]
	Pancreatic adenocarcinoma	Upregulated	Tissue	[30]
Urinary system	CC	Upregulated	Tissue	[31]
	PC	Upregulated	Tissue and cell	[32]
Other	HNSCC	Upregulated	Tissue	[33]

Abbreviations: GC—gastric cancer; CRC—colorectal cancer; LSCC—laryngeal squamous cell carcinoma; CC—cervical cancer; PC—prostate cancer; HNSCC—head and neck squamous cell carcinoma.

## 3. Clinical Prognostic Value

As paxillin expression is more widely studied in cancer, the clinical significance of paxillin has received increased amounts of attention. The aberrant expression of paxillin is often associated with carcinogenesis and is associated with poor tumor prognosis (Table 2), so the assessment of paxillin expression may provide some promising value in the prognosis of cancer.

Abnormal paxillin expression is associated with many clinicopathological parameters such as aggressiveness, distant metastasis, and clinical stage in various tumors, including colorectal cancer [34], prostate cancer [35], salivary adenoid cystic carcinoma [36], and cervical cancer [31]. The study found that the expression of paxillin in squamous cell/squamous carcinoma (SC/ASC), a rare subtype of gallbladder carcinoma, was associated with high tumor lymph node metastasis, tumor invasion, and the overall survival of patients. Paxillin is an independent risk factor for poorer survival in SC/ASC patients [37]. Similarly, the high expression of paxillin in human laryngeal squamous cell carcinoma (LSCC) is associated with histopathological grade and tumor metastasis and can be used as an independent prognostic factor and an independent predictor of overall survival in patients [29]. Additionally, paxillin is phosphorylated by the FAK/Src kinase complex to generate p-paxillin. The high expression of p-paxillin in mobile tongue squamous cell carcinoma is associated with higher tumor invasiveness, metastasis, poorer disease-free survival, and overall survival time [38]. On the other hand, the FAK-Src-paxillin protein system, consisting of FAK, Src, and paxillin proteins, is associated with an aggressive phenotype in adult malignancies and increases mortality in children with neuroblastoma. The FAK-Src-paxillin system is recognized as a marker of poor prognosis in human neuroblastoma patients [39].

As a highly expressed central gene in glioblastoma (GBM), paxillin expression is negatively correlated with the overall survival, progression-free survival, and disease-free survival of GBM patients, which can be used as an independent prognostic factor and an independent prognostic biomarker in GBM patients [40,41]. The phosphorylation of paxillin at serine 178 by JNK to form pS178-paxillin is also considered to be a potential reliable prognostic marker for hepatocellular carcinoma [42]. In addition to the above description that paxillin is highly expressed in tumors, paxillin is also overexpressed in precancerous lesions with different histological morphologies. Notably, paxillin overexpression is not a specific biomarker for identifying precancerous lesions in high-risk patients [43].

Despite the fact that the role of paxillin in cancer has been studied for many years, many studies have also demonstrated its value as a prognostic factor in cancer patients, and this topic remains controversial. For example, paxillin overexpression is associated with tumor aggressiveness, poor tumor differentiation, and lymph node metastasis in LSCC, and paxillin is a predictor of poor survival and recurrence-free survival. However, it is not an independent predictor factor of LSCC [44]. Similarly, paxillin expression is associated with survival and metastasis in colorectal cancer patients, and this may be a potential predictor of metastasis and an independent prognostic factor for recurrence but not for survival [45]. Andisheh et al. also reported that paxillin expression could not distinguish benign salivary gland tumor cases from malignant salivary gland tumor cases, and paxillin expression was not associated with clinicopathological parameters in salivary gland tumor patients [46]. In addition, the expression of paxillin in esophageal squamous cell carcinoma was confirmed by immunohistochemistry to be higher than that in adjacent non-tumor cells, but no correlation was found between the expression of paxillin and the overall survival rate of patients, and paxillin was not an effective prognostic marker for patients [47].

Hence, the correlation between paxillin and clinicopathological features is not completely consistent between different tumors, suggesting that paxillin alone has a limited clinical prognostic value in tumors. Compared with paxillin alone, the combination of paxillin and other tumor markers can be a better choice for tumor prognosis.

**Table 2:** The prognostic value and clinical significance of paxillin in different cancers.

Tumor Type	Sample Size	Prognostic Value	Clinical Significance	Reference
GC	239	Independent prognostic factors for patient survival	Positively related to distant metastasis and advanced tumor stage	[7]
CRC	242	Independent prognostic factors for patient survival	Positively related to tumor histological grade, tumor size, clinical TNM stage, and distant metastasis	[34]
PC	386	Independent risk factors for lymph node metastasis in prostate cancer	Positively related to preoperative prostate-specific antigen levels, clinical tumor stage, lymph node metastasis, and seminal vesicle invasion	[35]
CC	430	-	Positively related to tumor stage, poor differentiation, lymphovascular space invasion, and lymphovascular metastasis	[31]
SC/ASC	46	Independent prognostic factors for patient survival	Positively related to larger tumors, higher TNM stage, lymph node metastasis, and tumor aggressiveness	[37]
LSCC	84	Independent prognostic factors for overall patient survival	Positively related to histopathological grade, lymph node metastasis, and TNM stage	[29]
TSCC	48	Independent factors of survival in disease-free patients	Positively related to tumor differentiation, disease-free survival, lymph node metastasis, and depth of invasion	[38]
GBM	325	Independent prognostic factors for overall survival of patients	Positively related to tumor grade and overall survival of patients	[41]
HNSCC	518	Independent prognostic factors for patient survival	-	[33]

Abbreviations: GC—gastric cancer; CRC—colorectal cancer; PC—prostate cancer; CC—cervical cancer; SC/ASC—squamous cell/adenosquamous carcinoma; LSCC—laryngeal squamous cell carcinoma; TSCC—tongue squamous cell carcinoma; GBM—glioblastoma; HNSCC—head and neck squamous cell carcinoma. TNM—tumor–node–metastasis.

## 4. Paxillin Expression and Tumor Cell Metastasis

### 4.1. Paxillin Regulates Tumor Cell Migration

Cell adhesion and migration are the cellular processes commonly used by tumor cells to invade surrounding tissues and metastasize. Tumor metastasis is one of the leading causes of human cancer death since epithelial tumors form in the normal tissue stroma and migrate outward in the tissue stroma in two modes of single-cell migration and collective migration [48]. However, these two migration patterns differ, with single-cell migrating cancer cells undergoing epithelial–mesenchymal transition (EMT), a loss of cell-to-cell contact [49], while collectively migrating cancer cells that maintain cell-to-cell junction integrity, experiencing only partial EMT [50].

The cyclic regulation of FA is a key step in the control of cell adhesion and motility. Paxillin and FAK are key components of focal adhesions and changes in their expression or localization and their phosphorylation status are often associated with cancer cell metastasis [51]. Studies have shown that pS178-paxillin can promote the reorganization of FA to promote the migration of pancreatic tumor cells [52]. At the same time, focal adhesion is an essential structure for cell–cell adhesion and cell–ECM connection, and its number also represents the potential of cell metastasis [53]. Lysophosphatidic acid modulates the tyrosine phosphorylation of FAK and paxillin and their relocation and induces the rearrangement of the actin cytoskeleton and focal adhesion structures to inhibit the migration of pancreatic cancer cells [54]. The cargo receptor c-Cbl targets the autophagosome for the degradation of paxillin phosphorylated at the Y118 residue, triggering FA breakdown, thereby regulating cell–matrix adhesion turnover and cell motility in breast cancer cells [55]. Retinoic acid can dissociate phosphorylated paxillin from FA to the nucleus to translocate and inhibit the formation of FA complex, which inhibits the adhesion and migration of breast cancer cells [56]. 17β-estradiol has also been reported to inhibit the migration of gastric cancer cells by inhibiting CCL-5 and the IL-6-induced phosphorylation of Src, Cas, and paxillin in human gastric cancer cells [57,58]. Mechanical stress-induced autophagy can promote the migration ability of Hela cells by enhancing the expression of paxillin [59].

It is currently believed that the Rho family of GTPases is involved in the migration and invasion of cancer cells, and the expression and activation of RhoA and ROCK affect the formation of actin stress fibers [60]. Paxillin is involved in coordinating the activation of the Rho family GTPases, thereby participating in various signaling pathways related to cell migration [61,62]. The phosphorylation of paxillin at Tyr 31 and Tyr 118 residues has been reported to activate the RhoA/ROCK pathway to regulate tumor cell migration and invasion [14], while many studies have also shown that the increased phosphorylation of paxillin at Tyr31 and Tyr118 residues is considered a marker of metastasis [3]. Furthermore, the inhibition of paxillin phosphorylation inhibits esophageal cancer cell migration by inhibiting Rac1 prenylation and reducing its activation [63]. Paxillin can regulate the integrity and contractility of E-cadherin-dependent junctions by controlling the balance of RhoA and Rac1 activities to regulate the integrity of cell–cell adhesion junctions, thereby maintaining the migration of breast cancer cell populations [64].

In addition, paxillin can also promote cancer cell migration by regulating the EMT process. Since EMT is involved in the process of the lysis of cell–cell adhesion, the loss of apical–basolateral polarity, and the reorganization of the cytoskeleton, it is believed that EMT plays a key role in cancer cell metastasis [65,66]. E-cadherin has a potential paxillin-binding sequence, so the binding of paxillin to cadherin may be direct. Furthermore, the knockdown of paxillin in HeLa cells has been shown to impair N-cadherin-mediated adhesion, thereby reducing the metastatic ability of cells [67]. Meanwhile, paxillin may provide an essential link in the FAK-dependent survival pathway, which contributes to antianoikis, thereby promoting EMT and tumor metastasis [68,69]. Moreover, the downregulation of paxillin also reduces the activation of extracellular regulated protein kinase (ERK) and inhibits the EMT process, thereby inhibiting colorectal cancer metastasis [70]. Another study also showed that reducing the expression of FAK and its downstream reactive protein paxillin inhibited the EMT process in melanoma cells, thereby inhibiting the migratory activity of tumor cells [71]. Therefore, the dysregulation of paxillin in tumor cells may lead to a reduction in cell–cell adhesion and inhibit the metastatic ability of cancer cells. Paxillin may be an important regulator of cancer metastasis.

### 4.2. Paxillin Regulates Tumor Cell Invasive Metastasis

Tumor cells can also utilize adherent structures called invadopodia, which contributes to the invasive metastasis of tumor cells by crossing the basement membrane. The dynamics of invadopodia are regulated by the phosphorylation of paxillin, which accumulates in the center of the invadopodia ring and may lead to the disassembly of the invadopodia core at the inner edge of the ring, resulting in the complete disassembly of the invadopodia. Since paxillin is also a major component of invadopodia/podosomes, paxillin is involved in invadopodia ring expansion to trigger the invasive migration of cells [72]. The downregulation of paxillin resulted in a reduced invasion of primary breast tumors and made tumor organoids less invasive in 3D collagen cultures [64], whereas the overexpression of paxillin made cancer cells more aggressive [33,73]. The inhibition of FAK-paxillin interaction may be an effective strategy to inhibit invadopodia-mediated matrix degradation to inhibit the invasion and metastasis of melanoma cells [74]. Likewise, the inhibition of the FAK-paxillin pathway can also inhibit the invasion of other cancer cells [75,76].

Some kinases and regulatory proteins can promote the phosphorylation of paxillin to elevate cancer cell invasion. The phosphorylation of paxillin by kinases such as the Src family enables breast tumor cells and metastatic breast tumor cells with a basal-like phenotype to exhibit enhanced invasiveness in confined spaces [77]. Sphingosine kinase 1 may promote the invasive metastasis of colorectal cancer by driving autophagy to induce paxillin expression and its phosphorylation [78]. Fibronectin promotes the phosphorylation of paxillin (tyr118) to promote gastric cancer cell invasion [79].

In addition, actin is an important part of the cytoskeleton, and the filaments (F-actin) formed by actin are one of the basic elements of the eukaryotic cytoskeleton, and F-actin is involved in maintaining cell shape characteristics, aspects of cell motility, and other processes. The actin network, made up of actin filaments, can provide mechanical support for cells and help cells perform signal transduction and material transportation. At the same time, the assembly and disassembly of the actin network is the source of power for cell movement [80]. Cancer cells undergo a series of events to metastasize, such as shape changes, cell adhesion, cell movement, and invasion, which allow cancer cells to form new tumors elsewhere [81]. Moreover, the reorganization of the actin network is required for cancer cell invasion and metastasis, and this process is regulated by various proteins, such as FAK, Src, and paxillin, the most common signaling components in F-actin reorganization [82,83,84]. Norcantharidin disrupts F-actin reorganization by inhibiting the FAK/paxillin axis, which inhibits cancer cell invasion [85]. As a breast cancer tumor suppressor, interferon-inducible protein IFIX overexpression can stabilize the cytoskeleton through various cytokeratins and downregulate paxillin to inhibit the invasiveness of human tongue squamous cell carcinoma in vitro [86].

Many studies have shown that adhesion during tumor cell invasion depends mainly on the cell adhesion molecules of the integrin family [87,88]. The overexpression of integrins, especially those containing the β1 subunit, promotes tumor cell migration and invasion [89]. Paxillin can associate with integrin cytoplasmic chains α4 and β1, and β1 integrin is a known marker of invasiveness. Oleanolic acid derivatives can downregulate the expression of integrin β1/FAK/paxillin by reducing the level of β1 integrin, thereby inhibiting the invasion and migration of breast cancer cells [90]. Additionally, the knockout of paxillin is thought to have different effects on different cell lines. In the prostate cancer cell line PC-3 osteolytic model and the mixed osteolytic/osteogenic LNCaP model, paxillin knockdown resulted in reduced matrix adhesion and a general increase in cell invasion, but the opposite occurred in the MDA-MB-231 breast cancer model. Therefore, paxillin is considered to have potential specific effects in different cell lines or cancer types [51].

## 5. Paxillin Affects Tumor Cell Survival and Angiogenesis

### 5.1. Tumor Cell Survival

Due to the strong adaptability of cancer cells to various environmental changes, compared with normal tissue cells, cancer cells are more able to survive and proliferate in different tissue microenvironments. This also facilitates the distant metastasis of cancer cells, and this metastatic potential exhibited by malignancies correlates with their ability to grow independently of anchorage [91]. Paxillin was found in earlier studies to promote anchorage-independent growth by interacting with the bovine papillomavirus type 1 (BPV-1) E6 oncoprotein [92]. The new study also shows that the interaction of cold-associated tyrosine phosphorylated protein 1 with paxillin is required for the anchorage-independent growth of cervical cancer HeLa cells [93]. Thus, paxillin can act as a regulator of the anchorage-independent growth of some cells.

Moreover, to some important extent, the abnormal expression of paxillin has an effect on the survival of cancer cells. The overexpression of paxillin promotes the proliferation of glioma cells [41], gastric cancer [7], cervical cancer [31], and colon cancer [94]. It has been reported that paxillin downregulation reduced the expression of cell cycle promoters cyclinD1, E2F1, and TYMS. Conversely, paxillin upregulation enhances several pro-proliferative pathways, such as CyclinD/Rb/E2F and DNA replication/repair pathways, which contribute to the abnormal proliferation of tumor cells [95]. In addition, paxillin can directly interact with the pro-survival proto-oncogene Bcl-2 to promote cancer cell survival [96]. Cell division protein regulator 1 enhances the proliferation of non-small cell lung cancer by regulating the FAK/paxillin signaling pathway to promote cell cycle progression [97]. Tumor-associated macrophages can be divided into two types, pro-inflammatory M1 and anti-inflammatory M2, each of which has different functions, and M2-type macrophages can promote tumor growth [98]. It has been reported that the polarization of M2 macrophages is associated with the malignant progression of colon cancer. Inhibiting the expression of paxillin in M2 macrophages can inhibit their polarization by reducing the proliferation ability of colon cancer cells [99,100]. Additionally, cancer cells can also use the integrin/paxillin control mechanism to release tumor necrosis factor-α (TNFα)-converting enzyme-containing vesicles to improve their own growth microenvironment [101].

### 5.2. Tumor Angiogenesis

In the process of tumor-induced angiogenesis, tumor cells secrete diffusible angiogenic factors to induce vascular endothelial cells to proliferate and migrate to form new tumor blood vessels, and the development of new blood vessels also contributes to tumor growth [102]. Tumor-secreted vascular endothelial growth factor (VEGF) increases capillary endothelial cell ingrowth by reducing paxillin expression to inhibit NRP2 expression in vitro and in vivo while stimulating the formation of new functional microvessels in vivo. The formation of good microvessels contributes to normal organ development, but the dysregulation of this process promotes malignancy [103]. VEGF receptor 2 (VEGFR2) acts as a major signal transducer in VEGF-regulated angiogenesis, and the inhibition of VEGF/VEGFR2/FAK/paxillin signaling inhibits tumor angiogenesis [104,105]. In addition, Minamiguchi et al. demonstrated that the downregulation of paxillin expression in human umbilical vein endothelial cells (HUVECs) could reduce the adhesion of HUVEC to vitronectin to inhibit tumor-cell-induced angiogenesis [102]. The signal transducer and activator of transcription 3 (STAT3) act as a marker of tumor angiogenesis, interacting with Src and forming a complex with paxillin. Paxillin deficiency leads to the mislocalization of Src-activated STAT3, while STAT3 activity upregulates VEGF expression to promote tumor angiogenesis [106]. FAK also regulates tumor angiogenesis and cell survival by forming complexes with phosphorylated paxillin and Src [107]. Then, the downregulation of paxillin expression regulates Src/FAK/STAT3 to inhibit angiogenesis in estrogen-receptor-positive (ER+) breast cancer [108].

It has long been believed that neovascularization derived from vascular endothelium is the only way for tumors to obtain blood supply. In 1999, Maniotis et al. first proposed a new tumor microcirculation model that is different from the classic tumor angiogenesis pathway and does not depend on the body’s endothelial cells. This is angiogenesis mimicry (VM) [109]. VM is a brand-new tumor angiogenesis mode that is different from the traditional tumor angiogenesis pathway. In some highly invasive malignant tumors, tumor cells form a microcirculation pipeline composed of only tumor cells through their own deformation. These pipelines are connected with endothelium-dependent blood vessels to form a blood-transporting pipeline system, which establishes a new and special tumor microcirculation [110]. Usually, tumor growth and metastasis depend on efficient microcirculation, which consists of angiogenesis, angiogenic mechanisms, and VM. As VM signaling-related markers, ephrin type-A receptor 2 (EphA2), FAK, and paxillin overexpression in human gallbladder carcinoma GBC-SD cells promote tumor growth and VM [111]. However, as an inhibitor of VM in gallbladder cancer, norcantharidin can block the EphA2/FAK/paxillin signaling pathway to inhibit tumor VM formation [112]. Likewise, ERK/MMP and FAK/paxillin signaling pathways are considered key pathways regulating VM and angiogenesis [113,114]. The long non-coding RNA MALAT1 can promote gastric cancer VM and angiogenesis through the ERK/MMP and FAK/paxillin signaling pathways [115]. Thus, p-paxillin is a potential regulator of VM and angiogenesis.

## 6. The Signal Pathways and Regulatory Factors of Paxillin in Cancer

**Figure 2:**
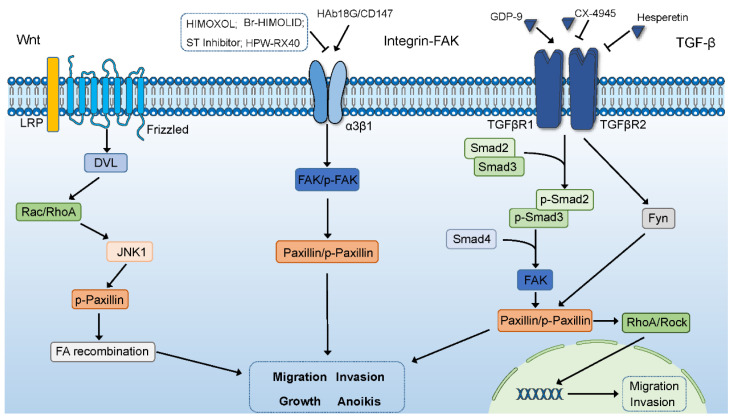
Paxillin participates in Wnt, integrin-FAK, and TGF-β signaling pathways. Paxillin induces FA recombination through the Wnt5a/JNK signaling pathway. HIMOXOL, Br-HIMOLID, ST inhibitor, and HPW-RX40 inhibit tumor progression by inhibiting the integrin-FAK signaling pathway to inhibit paxillin expression or phosphorylation level, while HAb18G/CD147 does the opposite. GDP-9 promotes paxillin expression and paxillin phosphorylation through Smad-dependent pathways. CX-4945 can reduce paxillin expression and paxillin phosphorylation by inhibiting the TGF-β signaling pathway. Hesperetin can inhibit TGF-β-mediated Fyn activation by reducing paxillin phosphorylation to inhibit RhoA expression and rock activation, which affects tumor migration and invasion.

**Figure 3:**
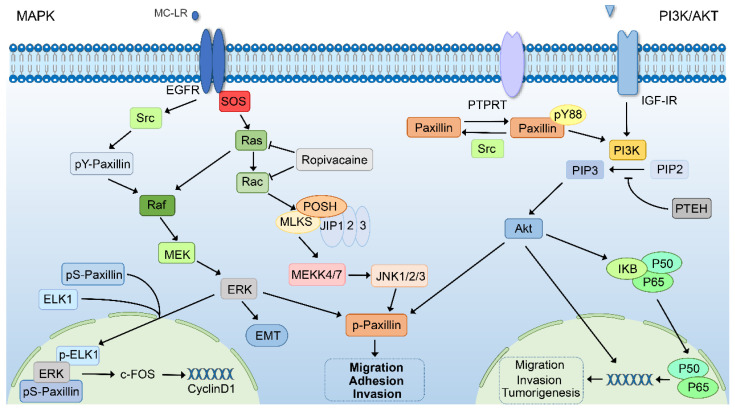
Paxillin participates in MAPK and PI3K/AKT signaling pathways. Paxillin is transferred to the nucleus through the MAPK pathway and promotes the combination of nuclear ERK and ELK1 to activate the expression of cyclin D1. Paxillin can also affect tumor cell EMT through the ERK signaling pathway. MC-LR promotes paxillin phosphorylation through the MAPK pathway. Ropivacaine reduces the activity of small GTPases, thereby inhibiting the MAPK/JNK signaling pathway to reduce the phosphorylation level of paxillin. GDP-9 promotes paxillin expression and paxillin phosphorylation through Smad-dependent pathways. CX-4945 can reduce paxillin expression and paxillin phosphorylation by inhibiting the TGF-β signaling pathway. Hesperetin can inhibit TGF-β-mediated Fyn activation by reducing paxillin phosphorylation to inhibit RhoA expression and rock activation, which affects tumor migration and invasion. IGF-IR increases paxillin phosphorylation by activating Akt and MAPK signaling cascades. PTPRT can dephosphorylate paxillin tyrosine-88 residue to inhibit the PI3K/AKT pathway and thus inhibit tumorigenesis. P50 and P65 can regulate the transcription and expression of paxillin, and PTEN can reduce the expression of paxillin by dephosphorylating PIP3 to inhibit the PI3K/AKT/NF-κB pathway.

In the development of tumors, genetic changes will occur in signaling pathways such as cell growth, apoptosis, and cycle progression, and the degree and mechanism of these changes vary with different tumors [116]. Many pathways are involved in the development of different stages of tumors, and many studies have also shown that paxillin participates in and activates a variety of signaling pathways to affect tumor progression (Figure 2 and Figure 3). At the same time, some non-coding RNAs and transcription factors can also affect tumorigenesis by regulating the expression of paxillin.

### 6.1. The Wnt Signaling Pathway

There are three Wnt signaling pathways, namely the Wnt/β-catenin signaling pathway, Wnt/Ca2+ pathway, and Wnt/PCP pathway. The Wnt/β-catenin pathway is a classic pathway, and the main ligands include Wnt1, Wnt3, and Wnt8b. After the ligands bind to the receptor Frizzled, they can lead to the accumulation of β-catenin in the cytoplasm. Unphosphorylated β-catenin, which accumulates to a certain extent, is transported to the nucleus, where it regulates the expression of target genes. The main ligands in other Wnt signaling pathways include Wnt5a, Wnt7a, and Wnt11. When the ligand binds to its receptor Frizzled, the Wnt/Ca2+ pathway activates phospholipase C to increase the amount of intracellular Ca2+, which activates Ca2+/calmodulin-dependent kinase II and calcineurin to regulate gene expression. The Wnt/PCP pathway activates the small GTPase Rho/Rac and JNK through the disheveled protein to regulate the expression of downstream target genes to affect cell movement [117,118]. Although different Wnt signaling pathways regulate different physiological processes in cells, the dysregulation of Wnt signaling pathways is associated with the progression of different tumors [119]. It has been reported that JNK can regulate cell migration by promoting the serine phosphorylation of paxillin. Wnt5a can promote the migration of pancreatic cancer cells by promoting paxillin phosphorylation through Wnt5a/JNK signaling [12,52].

### 6.2. The Integrin-FAK Signaling Pathway

When cells come into contact with the ECM, FAK, as one of the important signaling molecules of integrins, is recruited into focal adhesions and activated via phosphorylation during integrin-mediated adhesion. Activated FAK regulates the physiological or pathological process of cells by recruiting different signaling protein molecules to activate different downstream signaling pathways, such as proliferation, motility, and differentiation [120,121]. As a transmembrane glycoprotein in the immunoglobulin superfamily, CD147 is usually highly expressed in tumor cells. HAb18G is a liver-cancer-associated antigen that has the same nucleotide sequence as CD147. The overexpression of HAb18G/CD147 has been proven to promote the migration of liver cancer cells by increasing the expression of integrin α3β1 and activating the integrin-FAK signaling pathway to increase the expression of paxillin [53]. In addition, another report also showed that two synthetic derivatives of oleanolic acid (OA), HIMOXOL and Br-HIMOLID, can inhibit the expression and phosphorylation of FAK and paxillin through the integrin β1/FAK/paxillin signaling pathway, which can inhibit the migration and invasion of breast cancer cells [90]. Sialyltransferase (ST) has been shown to be associated with the malignant development of tumors, and its overexpression can enhance the migration, invasion, and adhesion of cancer cells [122,123,124,125]. Therefore, ST inhibitors inhibit breast cancer growth by inhibiting integrin expression and the phosphorylation of FAK and paxillin [126]. When the adhesion between cells and ECM is lost, cells are prone to anoikis, but some cancer cells can increase their own metastatic ability after acquiring anoikis resistance [127]. Compounds containing β-nitrostyrene have been shown to have multiple biological activities. As a derivative of 3,4-methylenedioxy-β-nitrostyrene, HPW-RX40 can not only inhibit integrin from preventing platelet aggregation but also inhibit the activation of the integrin β1/FAK/paxillin signaling pathway from enhancing the sensitivity of breast cancer cells to anoikis [128,129].

### 6.3. The TGF-B Signaling Pathway

The abnormal TGF-β signaling pathway can lead to the occurrence of various diseases such as tumors, cardiovascular diseases, and immune diseases. In addition, the TGF-β signaling pathway has two sides in the process of tumor development. It can not only play an inhibitory role in the early stage of the tumor but also play a promoting role in the late stage of the tumor [130,131]. The TGF-β pathway can be divided into the canonical signaling pathway (Smad-dependent pathway) and the non-canonical signaling pathway. In the canonical signaling pathway, ligands bind to TGF-β type I and type II receptors to activate Smad2/3, and then Smad2/3 forms a complex with Smad4 and transfers to the nucleus to regulate the expression of downstream target genes. In the non-canonical signaling pathway, the TGFβ receptor complex transmits signals through other factors such as P38, ERK, and JNK and indirectly participates in processes such as apoptosis, EMT, migration, proliferation, and differentiation [132,133]. Growth differentiation factor 9 (GDF-9) has been reported to promote the metastatic ability of prostate cancer cells by promoting the expression of FAK and paxillin through a Smad-dependent pathway [134]. Protein kinase CK2 (casein kinase 2) can regulate various signaling pathways to participate in cell physiological or pathological processes [135]. As an inhibitor of CK2, CX-4945 can inhibit the migration and invasion of lung adenocarcinoma cells by inhibiting the TGF-β signaling pathway to reduce the expression of FAK and paxillin [136]. The RhoA/ROCK signaling pathway is involved in TGF-β1-mediated tumor cell metastasis [137]. Proto-oncogene tyrosine–protein kinase Fyn is a member of the Src family kinases, and Paxillin is one of its substrate molecules. Fyn has been shown to be an important regulator of cell invasion and migration in various cancers [32,138]. Hesperetin can inhibit triple-negative breast cancer cell migration and invasion by inhibiting the TGF-β1-mediated Fyn/paxillin/RhoA signaling pathway [139].

### 6.4. The MAPK Signaling Pathway

The MAPK signaling pathway consists of three different levels of signaling molecules, namely mitogen-activated protein kinase kinase kinase (MAPKKK), mitogen-activated protein kinase kinase (MAPKK), and mitogen-activated protein kinase (MAPK). The process is that after the ligand binds to the receptor, MAPKKK, MAPKK, and MAPK are sequentially activated to regulate gene expression [140]. MAPK is a serine–threonine kinase that can be divided into four subgroups: ERK, P38, ERK5, and JNK. Therefore, there are four pathways in the MAPK signaling pathway, and the more classic pathway is the MAPK/ERK signaling pathway. Therefore, the MAPK signaling pathway is involved in various physiological processes such as cell proliferation, differentiation, apoptosis, and metastasis, but its abnormal activation also promotes the progression of various tumors [141,142]. Paxillin has been reported to regulate the MAPK/ERK signaling pathway in two ways. One is the phosphorylation of paxillin tyrosine by Src to regulate the activation of ERK1/2 specifically, and the other is the expression of downstream genes mediated by the downstream signaling molecules of ERK [143]. It has also been reported that paxillin translocates to the nucleus after MAPK-pathway-dependent serine phosphorylation to promote nuclear ERK and ELK1 binding, which induces the expression of c-FOS to activate cyclin D1 expression [144]. In addition, the downregulation of paxillin can inhibit the ERK signaling pathway to inhibit EMT in colorectal cancer cells by reducing the phosphorylation expression of ERK1/2 while keeping the total ERK1/2 level unchanged [70]. Microcystin-LR (MC-LR) promotes the adhesion ability of liver cancer cells by activating the MAPK/ERK1/2 signaling pathway to phosphorylate paxillin [145]. As a local anesthetic, ropivacaine can reduce the activity of small GTPase by inhibiting the prenylation of small GTPase, which can inhibit the phosphorylation level of paxillin by inhibiting the MAPK/JNK signaling pathway, thereby reducing the migration of esophageal cancer cells [63].

### 6.5. The PIK3/AKT Signaling Pathway

The PI3K/AKT pathway is activated in a variety of cancers and is involved in the process of cancer cell proliferation, survival, invasion, and metastasis and is considered a potential therapeutic target. Phosphatidylinositol kinase 3 (PIK3) is a dimer composed of regulatory subunit p85 and catalytic subunit p110, which catalyzes phosphatidylinositol 4,5-bisphosphate (PIP2) to phosphatidylinositol 3, 4, 5-triphosphate (PIP3). PIP3 can activate the downstream signaling molecules by activating the proto-oncogene Akt protein to regulate various physiological and pathological processes [146,147,148]. Insulin-like growth factor receptor I (IGF-IR) increases paxillin phosphorylation by activating Akt and MAPK signaling cascades to promote bladder cancer cell migration and invasion [149]. As the upstream kinase of paxillin, Src can directly phosphorylate paxillin tyrosine-88 residue, while the tumor suppressor protein tyrosine phosphatase receptor-T (PTPRT) can make paxillin tyrosine-88 residue dephosphorylation. At the same time, phosphorylation-Akt is the downstream substrate of pY88-paxillin, so knocking out the expression of PTPRT can phosphorylate paxillin tyrosine-88 residue to activate the PI3K/AKT pathway to promote colorectal carcinogenesis [26]. Notably, tumor suppressor protein phosphatase and tension homolog (PTEN) acts as a phosphatase that can dephosphorylate PIP3 to reduce Akt phosphorylation status and inhibit downstream signaling events regulated by Akt [150]. It is reported that P50 and P65 of the NF-κB subunit can regulate the transcriptional expression of paxillin. PTEN can reduce the expression of paxillin by inhibiting the PI3K/AKT/NF-κB pathway, which plays a role in inhibiting the migration and invasion of colon cancer cells. In addition, inhibiting the PI3k/Akt signaling pathway has also been shown to suppress the polarization of M2 macrophages by downregulating paxillin, thereby inhibiting the proliferation and invasion of colon cancer cells [151].

### 6.6. Other Regulatory Factors of Paxillin in Cancer

Current research shows that non-coding RNA plays an increasingly important role in the occurrence and development of various human diseases, and its dysfunction is closely related to cancer [152]. As one of the noncoding RNAs, long noncoding RNAs (LncRNAs) and microRNAs are involved in many physiological and pathological processes. miRNA can regulate gene expression by binding to the mRNA of the target gene, and lncRNA can interact with protein, RNA, and DNA to regulate gene expression [153,154]. Many studies have also shown that some non-coding RNAs or transcriptional regulatory factors can directly or indirectly regulate the expression of the paxillin gene to participate in the occurrence and development of tumors. The overexpression of miR-216b inhibits the proliferation, migration, and invasion of GC cells by partially regulating the expression of paxillin [155]. miR-212 acts as an epigenetically silenced tumor suppressor to suppress migration and invasion phenotypes of GC cell lines by downregulating paxillin expression [156]. Paxillin is one of the target genes of miR-137, and the overexpression of miR-137 can reduce the level of paxillin in colon cancer cells, which significantly inhibits cancer cell proliferation and metastasis [157]. It has been reported that lncRNA XIST, as a competitive endogenous RNA (ceRNA) of miR-137, is significantly upregulated in non-small cell lung cancer. LncRNA XIST, miR-137, and paxillin can form a ceRNA network to promote the proliferation and invasion of cancer cells [158]. Silencing lncRNA XIST can also promote gastric cancer cell apoptosis through the lncRNA XIST/miR-132/paxillin signaling axis [159]. At the same time, lncRNA DLX1-AS231, miR-199b-5p, and paxillin can also form a ceRNA network to regulate the proliferation, EMT, and cisplatin resistance of triple-negative breast cancer cells [160]. miR-218 and miR-27b can also regulate the occurrence and development of oral squamous cell carcinoma and colon cancer by targeting paxillin [161,162]. In addition, the carcinoembryonic splicing factor muscleblind-like-3 (MBNL3) splices the lncRNA-PXN-AS1 transcript containing exon 4 so that the lncRNA-PXN-AS1 transcript binds to the paxillin mRNA 3ʹUTR to avoid paxillin degradation, which increases paxillin expression to promote HCC progression [163]. As a transcriptional repressor, the CCCTC-binding factor (CTCF) can regulate the transcriptional inactivation of miR-137 to promote the expression of paxillin to promote EMT and radioresistance in esophageal squamous cell carcinoma [164]. ETS variant 4, a member of the ETS transcription factor PEA3 subfamily, was found to directly regulate the expression of the paxillin gene to induce the proliferation and migration of non-small cell lung cancer cells [165].

In summary, paxillin can directly or indirectly regulate Wnt, integrin-FAK, TGF-B, PIK3/AKT, and MAPK signaling pathways and participate in tumor invasion, metastasis, proliferation, apoptosis, and other processes. It can also be directly or indirectly regulated by some miRNAs, lncRNAs, and transcription factors to promote or inhibit tumor progression, which suggests that paxillin can be used as a target for antitumor drug development in the future.

## 7. Therapeutic Potential

Aberrant paxillin expression is associated with the malignant progression of cancer, so the modulation of paxillin expression may serve as a potential therapeutic target. Currently, certain drugs to treat cancer work by targeting paxillin or signaling pathways involving paxillin. Therefore, a further understanding of paxillin’s function may lead to the development of new cancer therapies developed against paxillin. Here, we will briefly discuss cancer therapeutics and potential drugs targeting paxillin as well as its signaling pathways (Table 3).

From the above, it can be seen that the FAK/paxillin signaling pathway is involved in several tumor-promoting pathways, so reducing FAK signaling by inhibiting FAK-paxillin interaction is considered a potential tumor therapy strategy. For example, a synthetic antibody-selective inhibitor designed against the paxillin LD2 and LD4 motifs can alter FA kinetics by inhibiting FAK-paxillin interaction by competing with FAK for the binding site of paxillin [166]. Furthermore, targeting FAK in cancer therapy may also inhibit paxillin-mediated functions. Paxillin can be a biomarker for FAK therapy [167]. Although the pharmacologically targeted development of FAK is in the first stage, the development of many small-molecule FAK inhibitors is at the stage of preclinical and clinical trials, but some small-molecule FAK inhibitors have achieved initial success in preclinical models [168,169].

Some other potential tumor therapeutic compounds can play a certain role in tumor therapy by targeting paxillin and its signaling pathway. For example, magnolol can inhibit the expression of lysyl oxidase from disrupting FAK/Src/paxillin signaling to inhibit the migration and invasion of breast cancer cells, which is considered to be an ideal strategy for breast cancer treatment [170]. A newly developed novel ST inhibitor, Lith-O-Asp, can inhibit lung cancer cell metastasis by inhibiting FAK/paxillin signaling and expressing antiangiogenic factors and is considered a potential antimetastatic therapy [171]. Mifepristone (RU486) is a synthetic steroid compound used as an abortifacient and has been used clinically in various tumor chemotherapies due to its anticancer activity [172,173,174]. Recent studies have further shown that mifepristone can inhibit the formation of the FAK/Src/paxillin complex to reduce the potential of breast cancer cell migration and adhesion [175]. A novel indole compound, SK228, was shown to disrupt the F-actin cytoskeleton and FAK/paxillin signaling axis in the sub-micromolar range to inhibit the growth of different lung and esophageal cancer cell lines [176].

In addition, some natural compounds are also involved in targeting paxillin and its signaling pathway in the process of assessing their antitumor abilities as they are expected to become potential anticancer drugs. For example, docetaxel inhibits the growth of prostate cancer cells and promotes apoptosis by inhibiting the phosphorylation level of paxillin [177]. The natural compound deguelin, as a potential antimetastatic drug in non-small cell lung cancer, exerts its antimetastatic effect by inhibiting FAK/Src/paxillin signaling by inhibiting the expression of cathepsin Z and its interaction with integrin β3 [178]. At the same time, Cucurbitacin B, which has strong anticancer activity, can inhibit the metastasis of breast cancer by inhibiting the phosphorylation of FAK and paxillin and destroying the FAK/paxillin signaling pathway [179].

However, tumor heterogeneity and resistance to chemotherapeutic drugs are the main reasons for tumor recurrence and poor patient survival. Tumor drug resistance is a limitation of many of the existing anticancer drugs, many of which are associated with paxillin. Cisplatin, as a commonly used anticancer drug, can effectively fight against a variety of tumors, but the chemoresistance generated in the process of medication limits the clinical application of cisplatin. It has been reported that phosphorylated paxillin increases Bcl-2 expression through the ERK signaling pathway as the cause of cisplatin resistance in non-small cell lung cancer, and Src or ERK inhibitors help restore the sensitivity of lung cancer patients to cisplatin chemotherapy [180]. On the other hand, inhibiting the activation of p-Erk in colorectal cancer cells by interfering with paxillin expression can increase the sensitivity of colorectal cancer cells to cetuximab [181]. Tyrosine kinase inhibitors have been shown to have significant therapeutic utility in non-small cell lung cancer; however, they also developed drug resistance during treatment due to paxillin-mediated ERK activation to reduce the interaction of Bcl2 with cell death mediators and to increase Mcl-1 expression [182]. In addition, 5-fluorouracil (5-FU) is a common chemotherapeutic agent for the treatment of colorectal cancer, but drug resistance often emerges later in treatment, resulting in poor treatment outcomes. The interaction of paxillin with Bcl-2 has been reported to be responsible for 5-FU resistance in colorectal cancer. Additionally, 5-FU combined with Src inhibitor (dasatinib), PAK1 inhibitor (IPA-3), or Bcl-2 antagonist (ABT-199) can overcome 5-FU resistance. As a result, the use of 5-FU in combination with Src inhibitors or Bcl-2 antagonists may improve the survival prognosis of colorectal cancer patients [183].

Therefore, gene therapy is now considered to be one of the best options to address the limitations of current treatments for the characteristics of chemotherapy drugs that have serious side effects and easily lead to drug resistance in cancer cells. To overcome the barriers of cell permeability and nuclease-mediated degradation, the use of pH-sensitive carbonate apatite nanocarriers as delivery vehicles for the targeted delivery of paxillin RNAi has become an effective potential therapy for breast cancer [184]. To explore the role of autophagy in breast cancer metastasis, researchers developed a tumor-activatable particle with an autophagy-inhibiting ability (named “D/PSP@CQ/CaP”). Because cell motility requires the assembly/disassembly cycle of focal adhesions, cells can regulate this process through autophagy. The potent tumor drug delivery ability of D/PSP@CQ/CaP can reduce the degradation of paxillin through autophagy inhibition to inhibit the autophagy-dependent breakdown of focal adhesions, thereby reducing the metastatic and growth ability of breast cancer [185]. Though the prospect of gene therapy is broad, there are few tumor gene therapy drugs for paxillin, and the gene therapy drugs developed for paxillin may become a potential treatment approach in the future.

All in all, further exploration of the paxillin pathway will help towards finding a solution to cancer metastasis and chemoresistance in the future.

**Table 3:** The potential tumor therapeutic drugs of targeting paxillin and its signaling pathway.

Drugs	Cancer Types	Functions	Mechanisms	References
Paxillin antibody selective inhibitor	-	-	Inhibition of FAK-paxillin interaction	[166]
Magnolol	BC	Migration, invasion	Inhibition of FAK/Src/paxillin signaling pathway	[170]
Lith-O-Asp	Lung cancer	metastasis	Inhibition of FAK/paxillin signaling pathway	[171]
Mifepristone (RU486)	BC	Migration, adhesion	Inhibits the formation of FAK/Src/paxillin complex	[175]
SK228	Lung and esophagus cancer	growth	Disruption of the F-actin cytoskeleton and FAK/paxillin signaling axis	[176]
Docetaxel	PC	Growth, apoptosis	Inhibits phosphorylation of paxillin	[177]
Deguelin	NSCLC	Metastasis	Inhibition of FAK/Src/paxillin signaling pathway	[178]
Cucurbitacin B	BC	Metastasis	Inhibition of FAK/paxillin signaling pathway	[179]
Hesperetin	BC	Migration, invasion	Inhibits phosphorylation of paxillin	[139]
D/PSP@CQ/CaP	BC	Metastasis, growth	Inhibition of autophagy-dependent degradation of paxillin	[185]

Abbreviations: BC—breast cancer; PC—prostate cancer; NSCLC—non-small cell lung cancer.

## 8. Conclusions

A growing body of research is supplementing our understanding of the potential role of aberrant paxillin expression in tumor cells and its impact on tumor malignant progression. According to the above summary, aberrant paxillin expression can regulate different biological functions such as tumor migration, heterotypic adhesion, invasion, survival, and angiogenesis in tumor malignant development by participating in different molecular mechanisms. In addition, the abnormal expression of paxillin in cancer patients is also closely related to the poor clinical prognosis of the patients. At the same time, there are more and more studies involving antitumor compounds and gene drugs targeting paxillin, some of which have achieved initial success in clinical trials. Consequently, paxillin may be a promising target for cancer therapy and prognosis in the future.

Though the detection of paxillin expression and the development of new oncology drugs targeting the paxillin gene are promising prospects for cancer clinical prognosis and treatment, many unanswered questions arise. For example, given the viability of paxillin as a single prognostic factor, many of the oncology therapeutics developed for paxillin are in the early stages of development, and there are still many uncertainties. Therefore, future research needs to fully combine paxillin with other prognostic factors to explore the relationship with poor clinical prognosis and to improve the sensitivity and specificity of paxillin in cancer prognosis. Therefore, a lot of basic research and clinical research is still needed to understand the function and therapeutic significance of paxillin fully. Furthermore, research on the drug treatment of paxillin and its molecular mechanism of drug sensitivity also needs to be conducted in the future. This will fully unleash the potential role of paxillin as a tumor therapy target in the future. A better understanding of the functional mechanism of paxillin will help us to understand tumor survival and metastasis processes and provide valuable opportunities for tumor therapeutic intervention in the future. Overall, this review highlights the role of the abnormal expression of paxillin in tumor progression and related molecular mechanisms, as well as the potential for paxillin to be used in tumor therapy.

## Data Availability

Not applicable.
